# Imaging findings of cesarean delivery complications: cesarean scar disease and much more

**DOI:** 10.1186/s13244-019-0780-0

**Published:** 2019-09-23

**Authors:** F. Rosa, G. Perugin, D. Schettini, N. Romano, S. Romeo, R. Podestà, A. Guastavino, A. Casaleggio, N. Gandolfo

**Affiliations:** 1 Department of Health Sciences (DISSAL), University of Genova, via A. Pastore 1, 16132 Genova, Italy; 2 Diagnostic Imaging Department, Villa Scassi Hospital-ASL 3, corso Scassi 1, Genova, Italy; 3 Diagnostic Imaging and Senology Unit, Policlinico San Martino, Largo R. Benzi 10, 16132 Genoa, Italy

**Keywords:** Cesarean delivery, Gynecology, Emergencies, Chronic cesarean delivery complications, Cesarean scar defect

## Abstract

In the last years, there has been a significant increase in the number of cesarean deliveries and, with it, of the number of complications following the procedure. They can be divided into early and late ones. We will illustrate herein the most common complications following cesarean section to help radiologists to recognize them. To familiarize with these various pathologic conditions is crucial to alert referring clinicians for a prompt and appropriate maternal and fetal management. Special attention will be given to the cesarean scar defect (CSD), the most common but also the most unknown of such conditions. Although often asymptomatic, a severe CSD represents a predisposing factor for subsequent complications especially in future pregnancies.

## Key points

- Early complications of caesarean-delivery.
- Late complications of caesarean-delivery.
- Detailed description of prevalence, clinical presentations, and imaging features of CSD.

## Background

The number of cesarean delivery is increasing and accounts for about one-third of all births both in the USA and in Italy [1]. The procedure is not free of peri- and postprocedural complications that can be divided into early and late ones [1]. Given cesarean delivery’s increasing use, there is also an increase of complications encountered.

Early complications include peri- and postprocedural conditions within 30 days after a cesarean delivery; late complication may occur also after some years and especially in a successive pregnancy (revision 2).

Overall early complication rate is about 14.5% and infection (such as endometritis and wound infections) is the most common complication. Fortunately, severe complications (i.e., uterine rupture) remain uncommon. Prolonged ruptured membranes, increased duration of labor prior to surgery but also anemia and obesity are considered risk factors for postoperative morbidity [2].

Familiarity with normal postprocedural findings of cesarean delivery (Table 1) is necessary to differentiate them from significant early complications such as hematomas, abscesses, wound infections, uterine dehiscence or rupture, and pelvic thrombophlebitis. In the immediate postoperative period, typical symptoms as fever, dropping hemoglobin level, unexpectedly heavy vaginal bleeding, and pain often motivate imaging studies. In this clinical scenario, ultrasonography (US) and computed tomography (CT) are the modalities of choice while the role of magnetic resonance (MR) is limited especially by its availability and acquisition time.

**Table 1:** Normal postprocedural findings [1]

Normal postprocedural findings of C-section	
Uterus dimension	Enlarge (average size is 9 × 12 × 14 cm)
Endometrial cavity	< 2 cm
Fluid in the uterine cavity	Normal (do not confuse it with infection or retained products of conception!)
Intracavitary gas	Possible findings in asymptomatic women up to 3 weeks postpartum (differential diagnosis with endometritis!)
Bladder flap hematoma	Normal if < 4 cm of diameters

Among late cesarean delivery complications, cesarean scar defect (CSD) is the most common but also the most neglected. In pregnant patients with a history of prior cesarean delivery, a severe CSD is a risk factor for both early (i.e., uterine rupture) and for late complications (i.e., ectopic pregnancy at the scar level and other scar-related abnormalities). For the evaluation of late complications US (if possible both transabdominal-US and transvaginal-US, TV-US), hysterography and MR imaging are the modalities of choice.

In this article, we will review the most common early and late complications of cesarean delivery and we will describe in detail the CSD in consideration of its high prevalence and its role as risk factor for the major part of the other caesarean delivery complications.

## Cesarean delivery complications

Complications of cesarean section (C-section) can be divided into early and late ones [1] as shown in Table 2.

- Early complications

**Table 2:** Acute and chronic caesarean delivery complications (adapted from [1])

Early complications	Late complications
Infections (most common) Endometritis, wound infection, abscess	Cesarean scar defect (most common)
Subfascial hematoma	Abdominal wall endometriosis
Bladder flap hematoma (> 4 cm)	Morbidly Adherent Placenta (placenta accreta, increta, and percreta)
Uterine dehiscence	Cesarean scar ectopic pregnancy
Uterine rupture	Cesarean scar retained products of conception

**Fig. 1:**
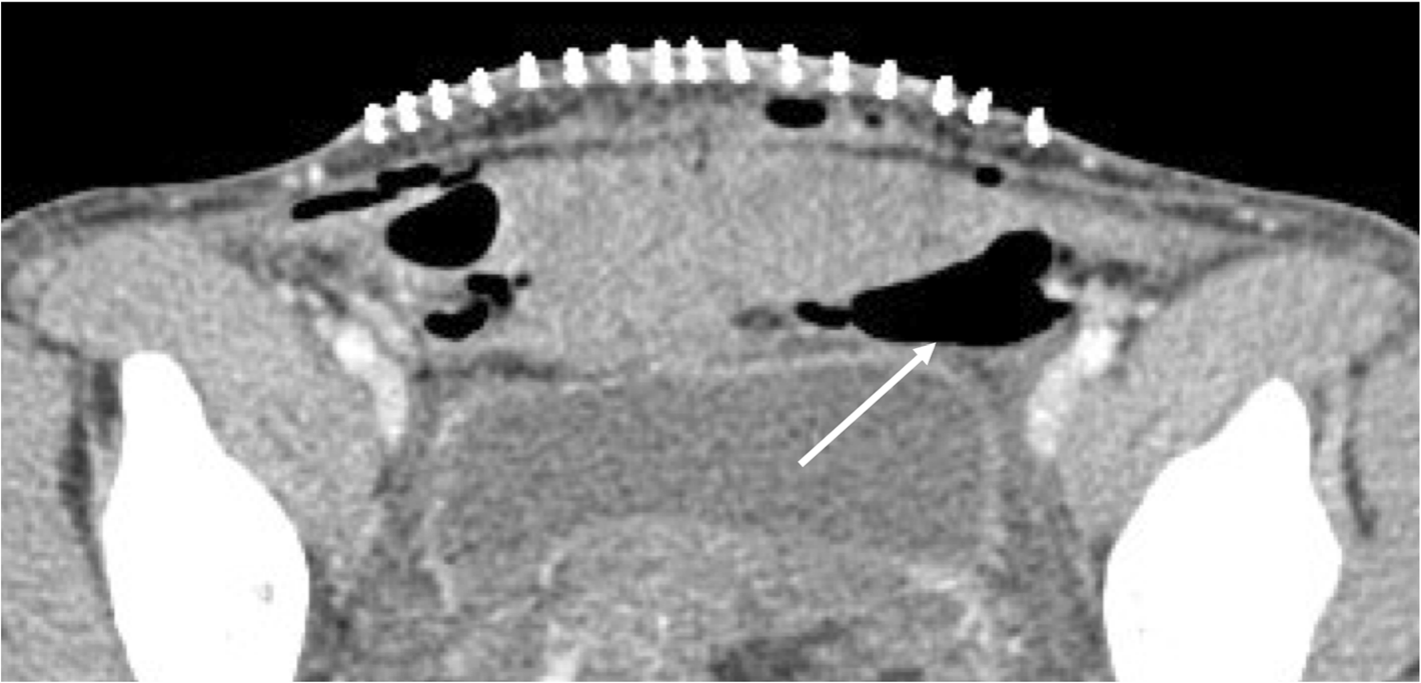
C-section wound infection: in the correct clinical scenario, inflammatory changes associated with gas within the soft tissues adjacent to the scar (white arrow) makes the diagnosis

The most frequent early complications are infections (such as endometritis, surgical wound infections, and abscesses) and hemorrhages (Fig. 1).

**Hemorrhage** can be due to lacerations of intra-abdominal (uterine and ovarian) or extra-abdominal arterial or venous vessels. They can be massive and life-threatening conditions.

**Fig. 2:**
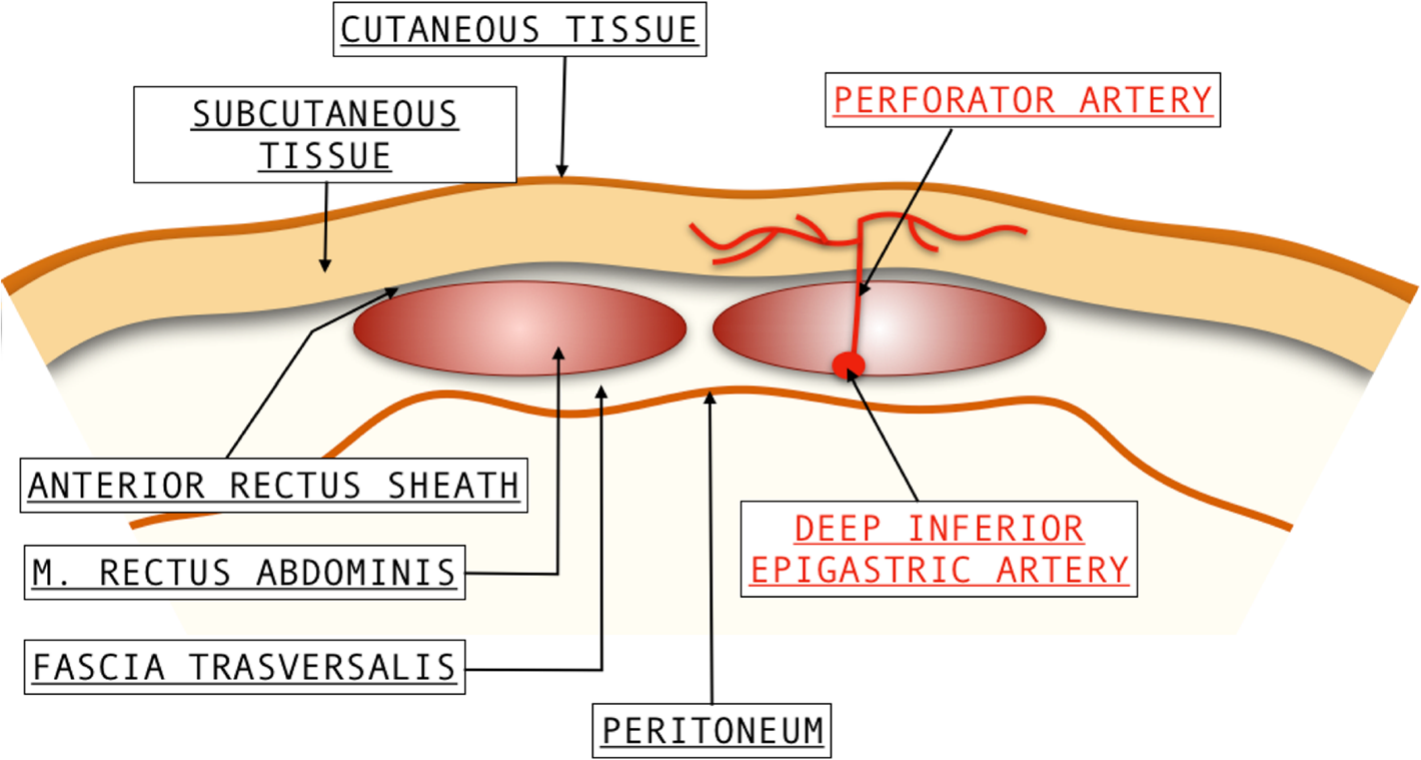
Extra-abdominals arteries or vein: inferior epigastric vessels lacerations can lead to rectus abdominis sheath hematoma or to subfascial hematoma

Extra-abdominal vessels laceration frequently involves the lower epigastric arteries (Fig. 2) and can lead to the formation of a hematoma within the rectus abdominis muscle (rectus sheath hematoma) or to an extraperitoneal hemorrhage with blood collection in the pre-vesical space, posterior to the rectus and transversalis muscles and anterior to the peritoneum (subfascial hematoma) [1].

**Fig. 3:**
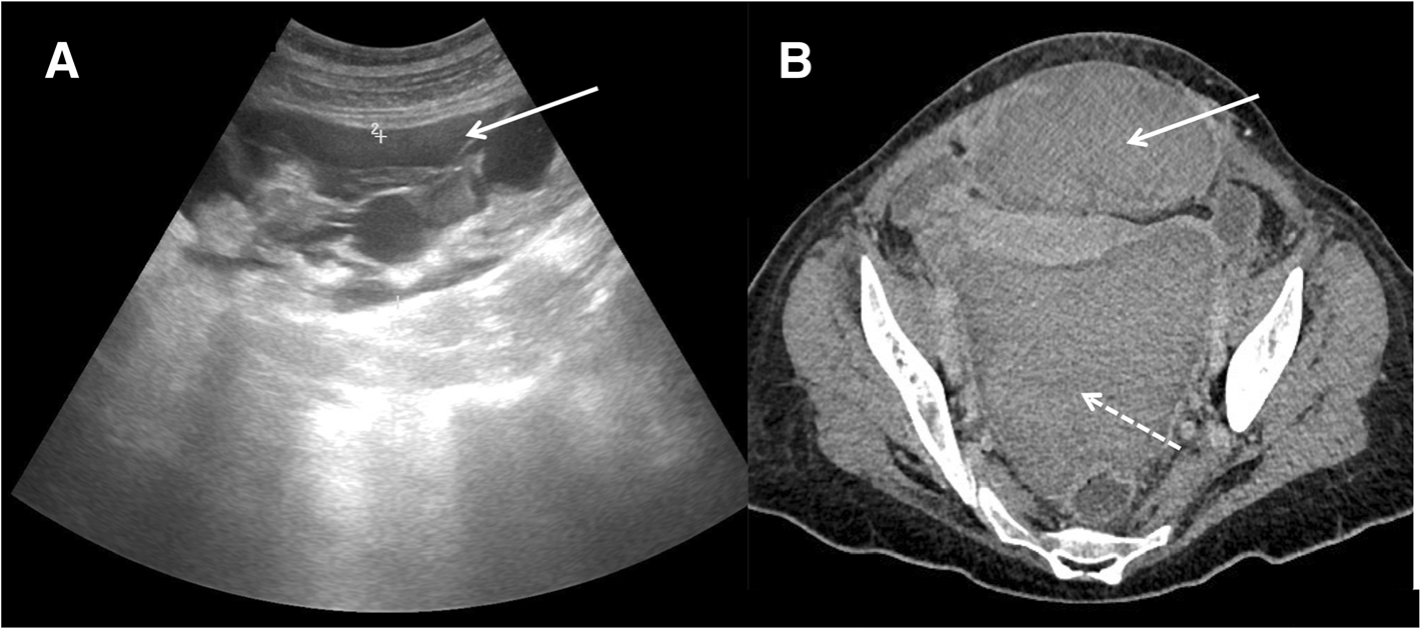
Subfascial hematoma associated with massive hemoperitoneum. **a** US examination showed a complex collection in this case in the rectus muscles (white arrow). **b** After contrast CT imaging confirmed the subfascial hematoma (white arrow) and showed also hemoperitoneum (dashed arrow)

**Fig. 4:**
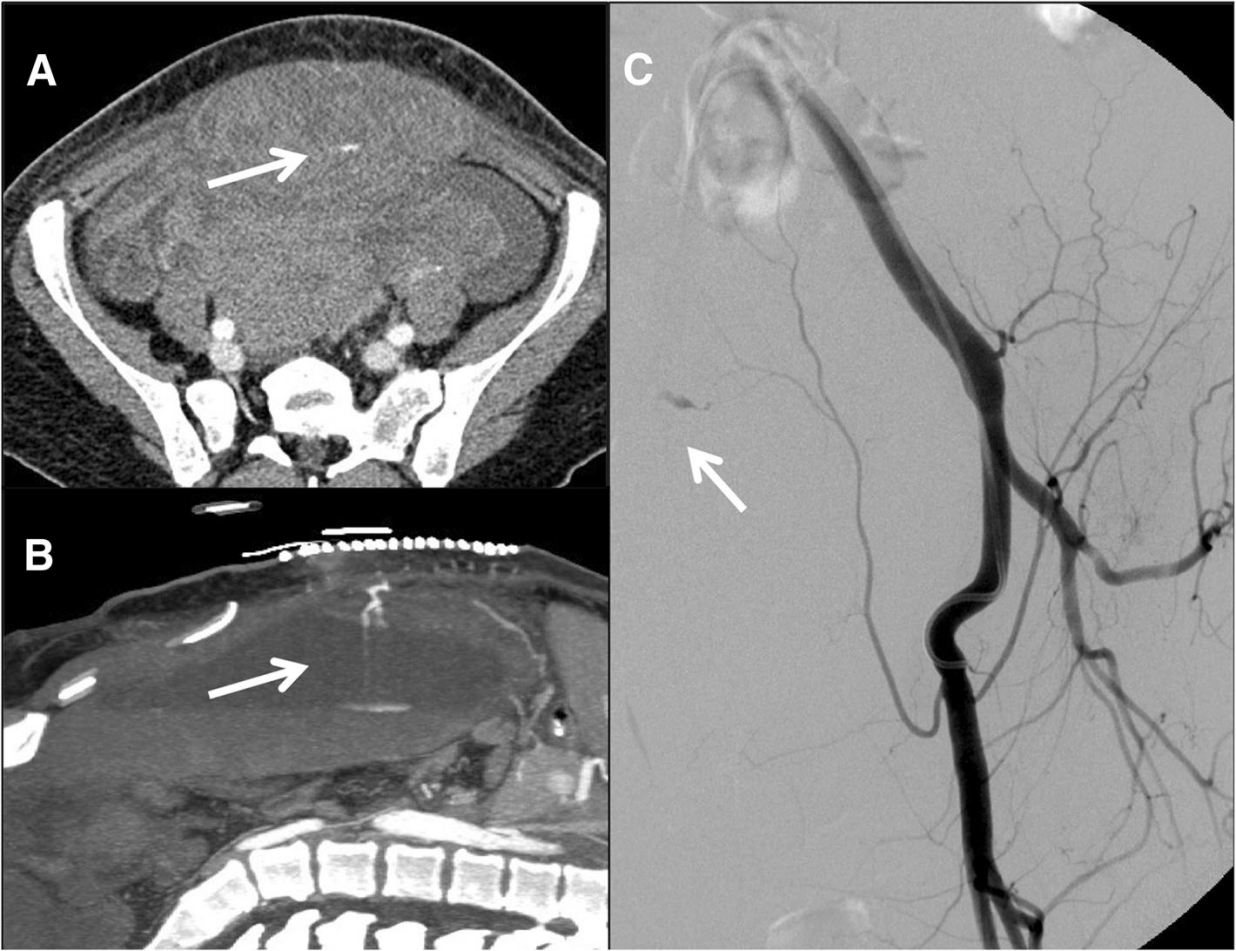
(Same patient of Fig. 3). Contrast-enhanced CT (**a**, **b**) showed contrast material extravasation (white arrow) suggestive for active bleeding confirmed by arteriography (**c**)

These two clinical entities can coexist and are rarely associated to hemoperitoneum (Figs. 3 and 4).

The so called “bladder flap hematoma” is located in the space between urinary bladder and lower uterine segment and its spread is limited by the overlying peritoneum [3, 4]. Small bladder flap hematoma can occur in up to 50% of the patients undergoing cesarean delivery with a low transverse incision and is considered a normal finding if < 4 cm [1]. Bladder flap hematoma larger than 5 cm is uncommon but it can be correlated to uterine scar dehiscence. Moreover, it can be a source of bacterial superinfection and, if large, it can spread through the broad ligaments into the retroperitoneum and into the peritoneal cavity with hemoperitoneum. For these reasons, the presence of a large bladder flap hematoma (> 4–6 cm) and sepsis unresponsive to adequate antibiotherapy would justify re-laparotomy. On US and CT, it is visualized as a hyperechogenic or hyperdense heterogeneous collection between the bladder and the inferior uterine segment; gas bubbles, internal septa, and peripheral vascularization are present in case of abscess formation. It is important to discriminate significant bladder flap hematomas from subfascial hematomas because only the first ones require incision of the peritoneum.

**Fig. 5:**
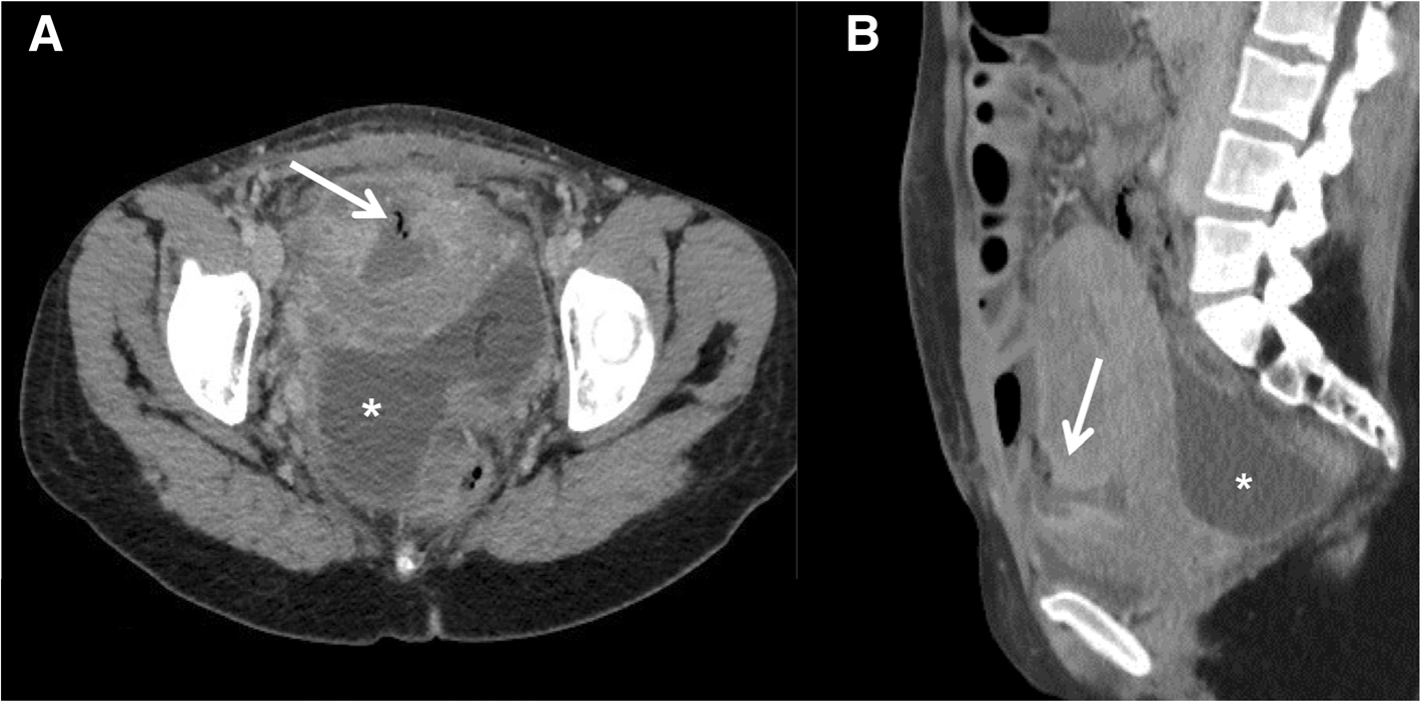
Uterine rupture**:** CT examination (**a**, **b**) showed the presence of gas within the uterine defect, extending from the endometrial cavity to the extra uterine parametrium (white arrow), in association with hemoperitoneum (asterisks). In the appropriate clinical setting, these features are highly suspicious for uterine rupture

**Uterine rupture** is the most severe early complication and is defined as the complete laceration of the uterine wall including its serous layer, creating a communication between the endometrial and peritoneal cavities with gas and blood leakage and consequent hemoperitoneum (Fig. 5). The incidence of uterine rupture among women with at least one prior CS was 0.5% and severe CSD is an important risk factor [5].

Partial rupture of the uterine wall, in which the serous layer remains intact, is called uterine dehiscence. The differential diagnosis between these two entities may be difficult [1].

“Red flags” for uterine dehiscence are the presence of a bladder flap hematoma > 5 cm and large pelvic hematomas. On the other hand, the presence of gas within the uterine defect, extending from the endometrial cavity to the extra uterine parametrium in association with hemoperitoneum is highly suspicious for uterine rupture. Demonstration of a continuous pathway between the endometrial cavity and the extrauterine collection, either by CT or MRI, is a pathognomonic finding for uterine rupture.

Due to the rarity of these conditions and the low correlation between radiological and surgical findings, there are not standardized diagnostic criteria. However, in an adequate clinical setting, these “red flags” can help radiologists at least to suspect a uterine rupture and to promptly guide patient management. Although uterine rupture is usually clinically recognized and managed by laparotomy, some cases with clinical indolent signs and symptoms may be more likely to be diagnosed with imaging. CT with multiplanar reformatted reconstruction can be considered a good initial imaging modality, due to its availability, rapid imaging acquisition time, and the possibility to use reformatted images, perpendicular to the plane of incision [1]. Nevertheless, MR could be superior to CT for the differential diagnosis between uterine dehiscence and rupture by delineating all uterine wall layers and identifying an intact serosa covering the myometrial gap [6]. Moreover, large hematoma or abscess usually associated with true dehiscence can be easily detected on MR. Differential diagnosis is important because uterine dehiscence can be managed conservatively instead uterine rupture require a surgical treatment.

- Late complications

As mentioned before, we will describe in detail the CSD due to its high prevalence and because it can be considered as a predisposing factor for the major part of the other cesarean delivery complications. Other common late complications are abdominal wall endometriosis, morbidly adherent placenta (MAP), cesarean scar ectopic pregnancy, and retained products of conception (RPOC) at the C-section scar level.

### Cesarean scar defect

The CSD is the most common complication after a cesarean delivery; it is reported with different nouns in literature (pouch, niche, or histhmocoele). It is defined as a focal thinning of myometrium or a dehiscence of the uterine scar, which appears with a triangular shape in continuity with the endometrial cavity [7].

CSD is considered severe if the incision depth is at least 50 or 80% of the anterior myometrium, or if the remaining myometrial thickness is ≤ 2.2 mm when evaluated by transvaginal ultrasound (US) [8].

- Risk factors

Risk factors to develop a CSD can be divided in non-modifiable and modifiable ones.

Non-modifiable risk factors can be mother-related (age, retroverted uterus) or labor-related ones (duration of labor > 5 h and cervical dilation at the time of delivery > 5 cm).

Modifiable risk factors are mostly related to the surgical technique (incision close to internal os, exclusion of endometrium during repair, single-layer closure) [8–11].

- Clinical symptoms

The exact prevalence of symptomatic CSD is difficult to quantify due to several factors such as heterogeneity of population studied, lack of knowledge about this problem, and the absence of accepted guideline criteria. However, it has been reported to range from 19.4 to 88% [12–14].

Severe complication, i.e., uterine rupture during a successive pregnancy, has an incidence of only 2% but this percentage increase up to 5% if the CSD is considered severe.

Clinical presentation is strongly heterogeneous, from absence of symptoms (most frequently) to presence of uterine bleeding, infertility, dyspareunia, and pelvic pain.

Abnormal vaginal bleeding is the most frequent symptom: a retrospective study showed that it is present in 76% of women with CSD. It is defined as a persistent vaginal bleeding from 2 to 12 days after the end of menstrual phase [15]. This bleeding is thought to be due to retention of blood within the defect cavity (niche) and its delayed emptying. Some authors consider the bleeding as due to in situ angiogenesis [16].

The mechanism of CSD-related infertility is not so clear: the main hypotheses are that retained blood or chronic inflammatory state can have negative influence on sperm transport and implantation [17, 18].

Etiology of chronic pelvic pain is related to the chronic inflammatory state associated with mucus-blood stagnation in the niche.

Lastly, a severe CSD, in women who desire another pregnancy, is considered as a risk factor for severe complications since it can be the site of ectopic pregnancy, placenta adhesive disorders, and uterine rupture.

Notwithstanding their high prevalence, CSDs are often undiagnosed. Since frequently asymptomatic, or with non-specific symptoms, they are no considered and not looked for. They are often diagnosed when abnormal findings are demonstrated during examinations done for other purposes.

Radiologists can help gynecologists, especially through MR examinations, to understand if symptoms are really CSD-related or due to other pathologic conditions and to individuate women with a severe CSD with a higher risk of severe complications.

- Radiological features

There are several imaging techniques to detect the CSD but there is no universal consensus about which is the *gold standard*. Moreover, there are no standardized diagnostic criteria.

**Fig. 6:**
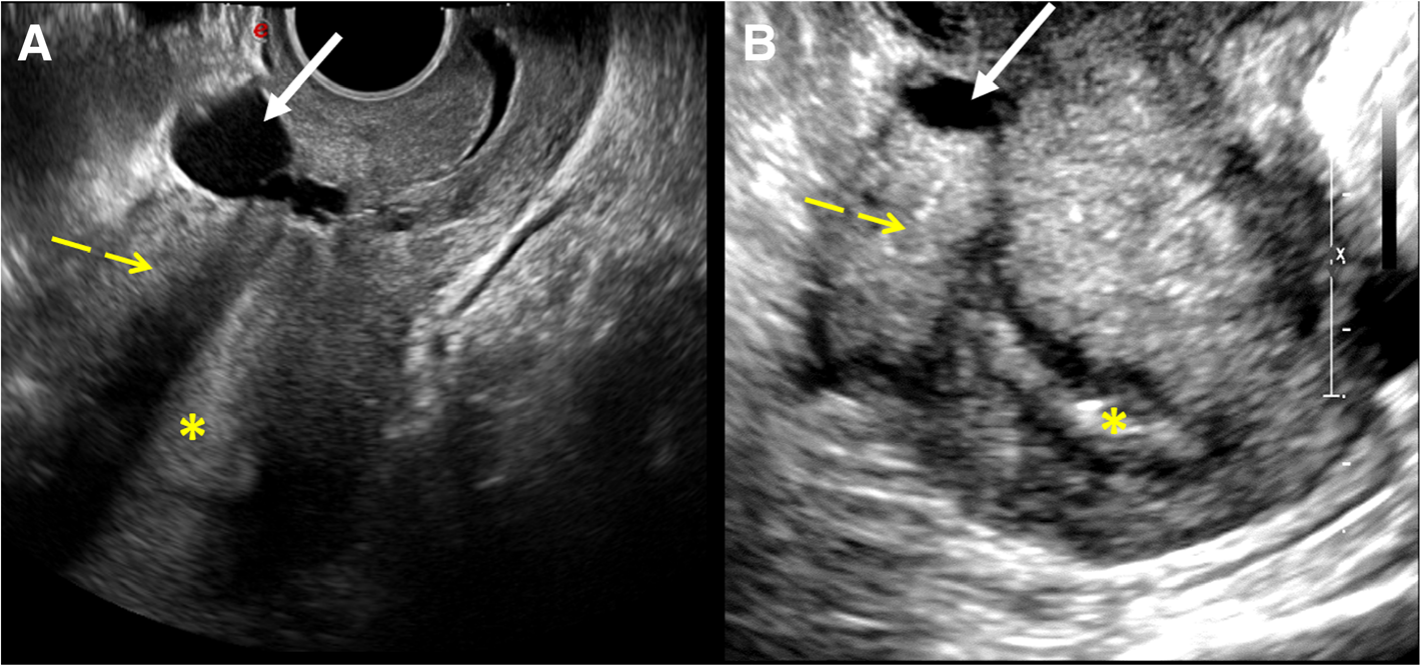
Cesarean scar defect: CSD appearance at TV-US examination (white arrow). It can look like a cystic lesion (**a**) or it can be an anechoic, triangular shape defect with apex pointing anteriorly, located at the anterior isthmus (**b**). Anterior uterine wall (yellow dashed arrows), endometrium (asterisks)

TV-US is a first level and widely used imaging technique. CSD is described as an anechoic, triangular shape defect with apex pointing anteriorly, located at the anterior isthmus. It can also look like a cystic lesion between bladder and lower uterine segment (Fig. 6). Differential diagnosis includes Nabothian cysts, prominent uterine vessels, and small leiomyomas.

The role of saline infusion sonohysterography is controversial: Osser at al. made a study of agreement between transvaginal sonographic findings with and without saline contrast enhancement [18, 19]. The agreement was good (percentage agreement varying from 88 to 100% and with Cohen’s kappa varying from 0.679 to 1.000). The authors concluded that CSD were better evaluated through saline contrast enhancement TV-US than with unenhanced ultrasound examination, because the demarcations of scar defects were more clearly delineated, more defects were detected, and more defects were classified as large at saline contrast-enhanced TV-US. These findings can be explained by possible washing away of mucus from the niche during saline infusion. So, some authors recommend this technique especially in the surgical planning [15–20]. However, it is more invasive, carries a small risk of complications (such as infections), and can overestimate the defect (about 1–2 mm) because of over-distention of the niche [20].

Hysterosalpingography is an imaging technique used to evaluate uterine cavity and tubal patency.

**Fig. 7:**
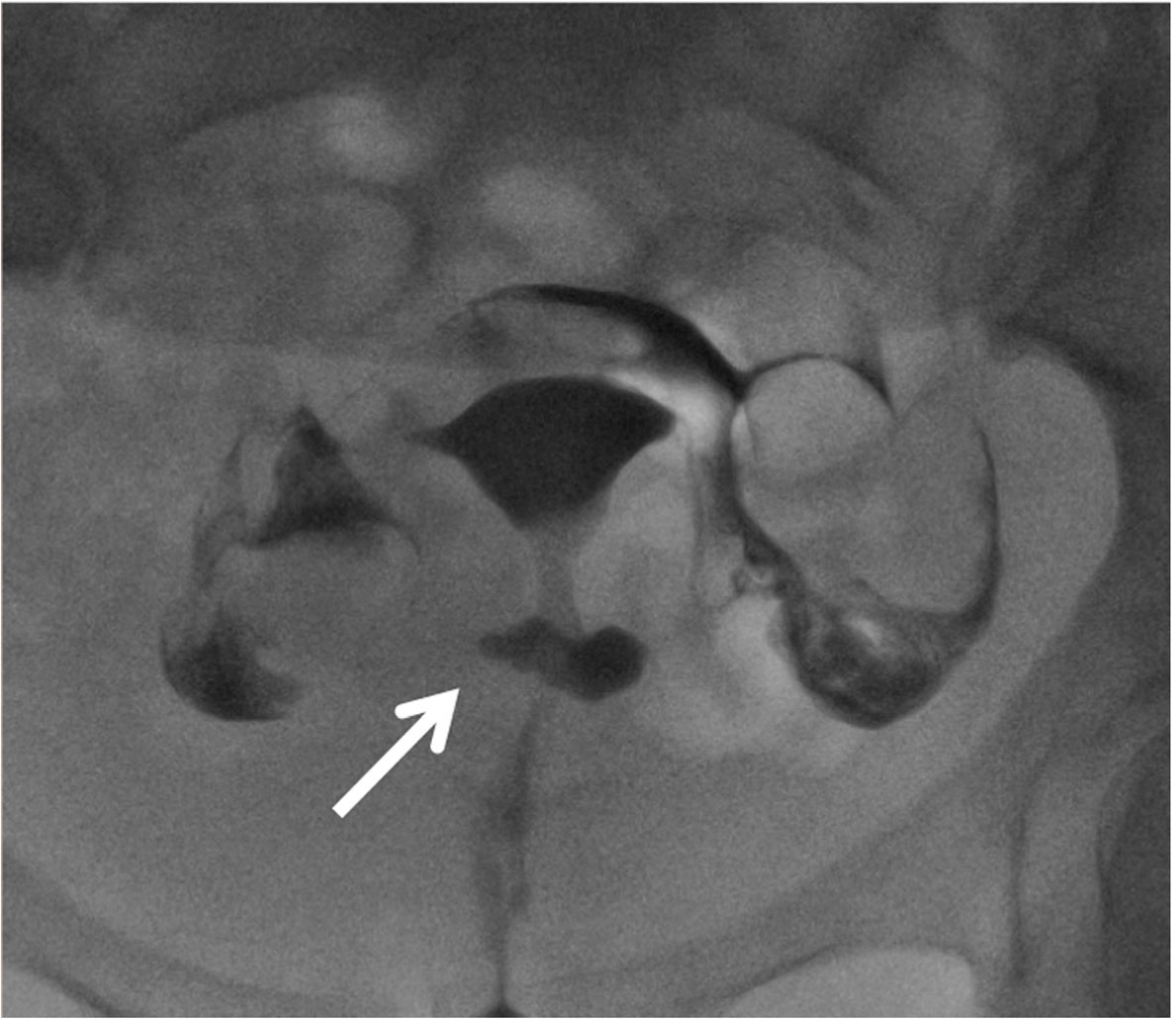
A 35-year-old woman underwent hysterosalpingography for infertility after a previous cesarean delivery CSD is detected as a leakage of contrast from endometrial cavity into a defect of the myometrium at the location of the previous C-section

**Fig. 8:**
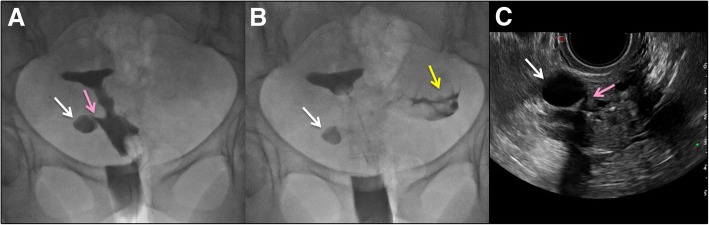
A hysterosalpingography (**a**, **b**) was performed for infertility after a previous cesarean delivery and right tubectomy due to a previous ectopic tubal pregnancy. **a**, **b** showed right antero-lateral istmocele (white arrow) in continuity with endometrial cavity (pink arrow), **b** demonstrated left normal intraperitoneal spill of contrast (yellow arrow). **c** Transvaginal US confirmed all findings and well demonstrated the continuity between the istmocele (white arrow) and the endometrial cavity (pink arrow)

Major indications are infertility, recurrent miscarriage, and evaluation of tubal ligation efficacy [21]. Hysterosalpingography can identify the CSD that can be the cause of secondary infertility after CS (Figs. 7 and 8). CSD is visualized as a leakage of contrast from the endometrial cavity into a defect of the myometrium at the location of a previous hysterotomy. Oblique views, with the patient leaning on her side, may better demonstrate the continuity between uterine cavity and the niche.

MR is a second level imaging technique. Due to its panoramic capabilities, it evaluates not only the lumen but also the uterine wall and allows an accurate differential diagnosis. Its role is fundamental to rule out other causes of symptoms like adenomyosis and leiomyomas. So, MR is especially useful in surgical planning, especially if other pathological conditions are present.

**Fig. 9:**
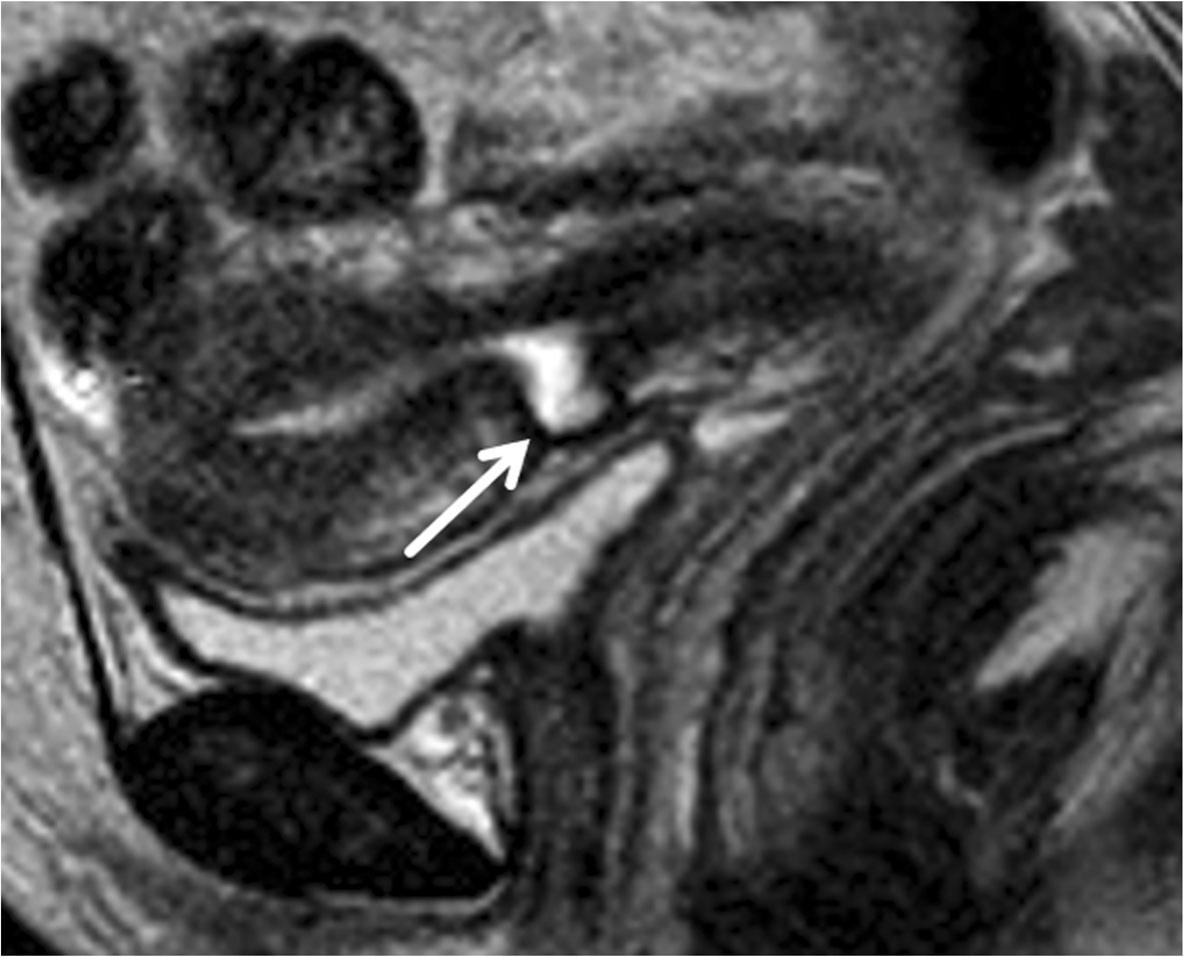
A 40-year-old woman underwent to MR for abnormal uterine bleeding. CSD is detected on T2WI as a myometrium defect with apex pointing anteriorly, located at the anterior isthmus (white arrow)

T2-WI (weighted imaging) clearly demonstrates the CSD with morphologic features analogue to the other modalities (Fig. 9).

- Classification

Most studies to evaluate CSD dimension and severity have been performed with transvaginal ultrasound and the same criteria can be also applied to MR [22]. The possibility to use the same classification systems makes easier the communication with gynaecologists.

**Fig. 10:**
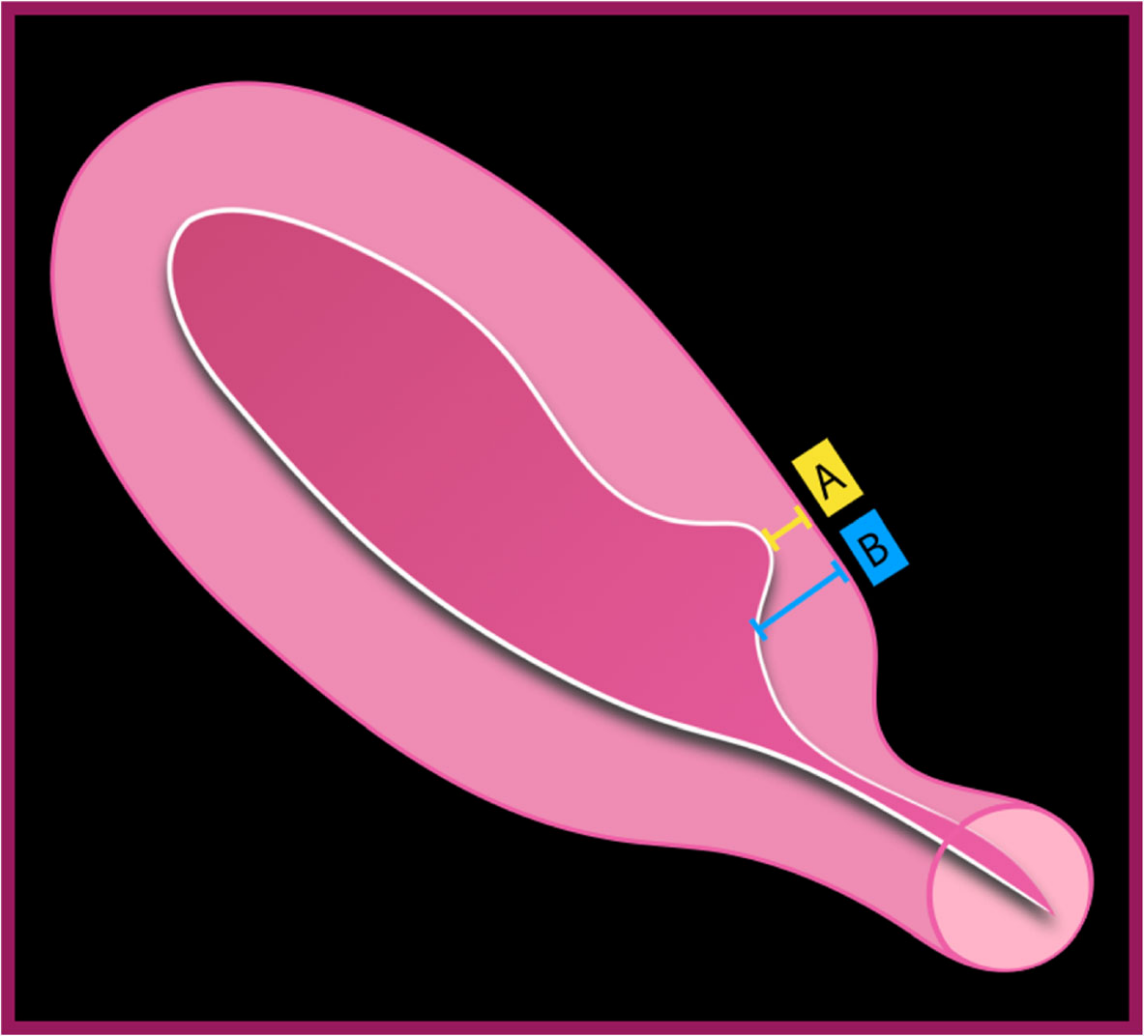
CSD classification and calculation of remaining **myometrium.** A = thickness of remaining myometrium; B = full-thickness adjacent to defect. Percentage of myometrium remaining: $x\left(\%\right)=\frac{\mathrm{A}}{\mathrm{B}}\ast 100$

The CSD severity is established through measurement of the ratio between myometrial thickness at the scar level and the thickness of adjacent myometrium: it is considered severe if the ratio is equal or inferior to 50% (Fig. 10) [8]. Another possible method is to use a cut-off of 2.2 mm for the remaining myometrium thickness at transvaginal US and a value ≤ 2.5 mm when the patient is evaluated by sonohysterography [8, 21].

It has been demonstrated that a ratio ≤ 50% correlates with symptomatic CSD [13, 18, 20, 23, 24] that are the only ones on which there is consensus about the need for treatment [19]. Imaging is crucial to rule out other causes at the basis of symptoms and to decide the most adequate treatment: from hormonal therapies to surgery with different approaches (laparoscopy, hysteroscopy, and vaginal procedure depending on the expertise of the surgeon). Furthermore, incidental and asymptomatic CSD must be always documented and reported, especially before gynecological procedures (evacuation, endometrial ablation, intrauterine device implantation) because of increased risk of complications (also fistula and abscess).

## Other late complications

**Abdominal wall endometriosis** is a rare event (incidence rate reported at 0.4% to 0.1%.); it is due to iatrogenic seeding of endometrial cells during hysterotomy that create a functioning endometrial tissue mass outside the uterine cavity [25]. It is a possible cause of painful abdominal mass in young women, classically with cyclic presentation. However, pain can be also constant.

**Fig. 11:**
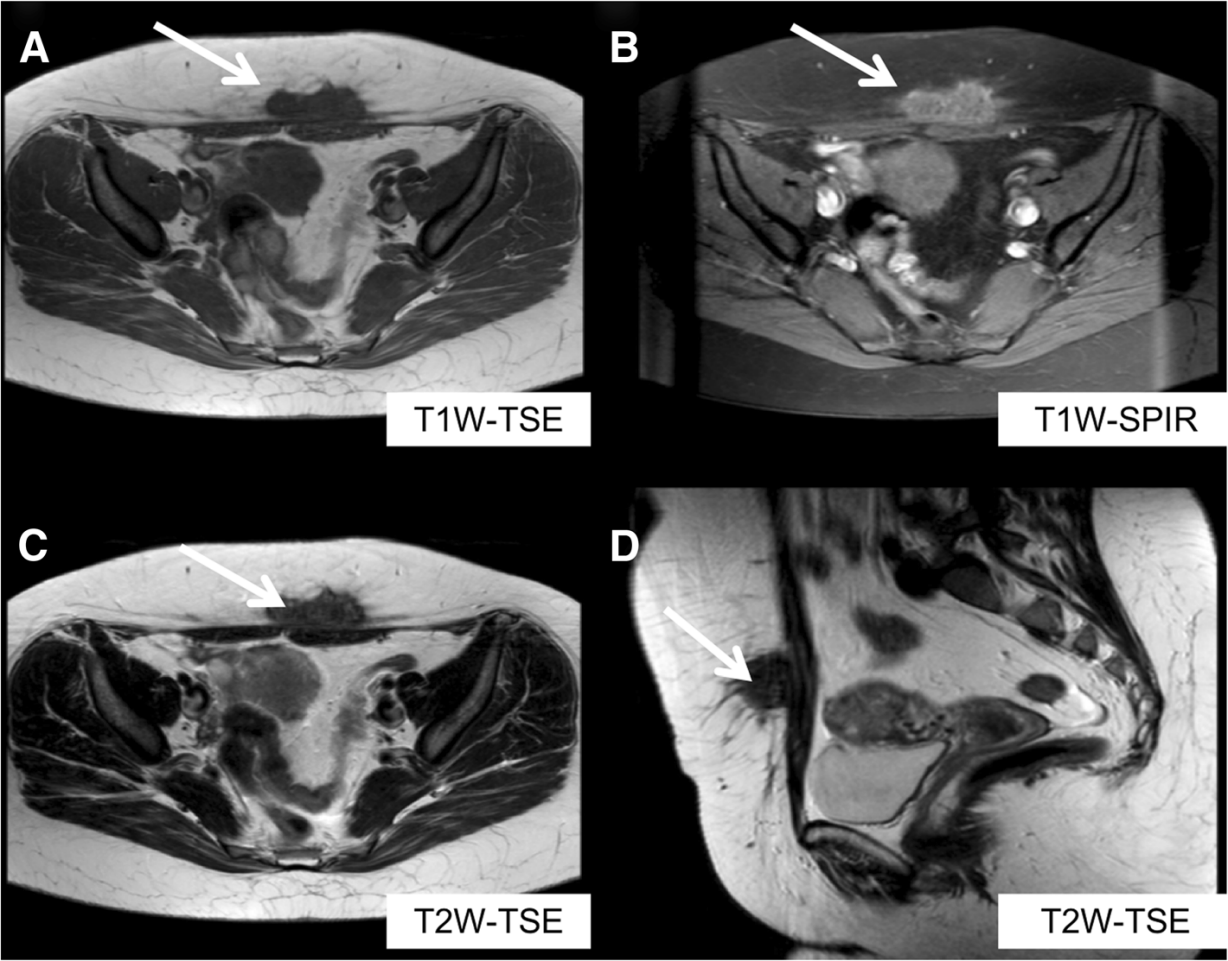
Abdominal wall endometriosis: this is a case of a women with abdominal wall palpable mass within incisions after cesarean delivery. MR imaging showed a solid nodule (white arrow) with low signal both on T1-TSE-WI (**a**) and T2-TSE-WI (axial, **c** and sagittal sections, **d**) with an iso-hyperintense signal on T1-SPIR WI (**b**)

**Fig. 12:**
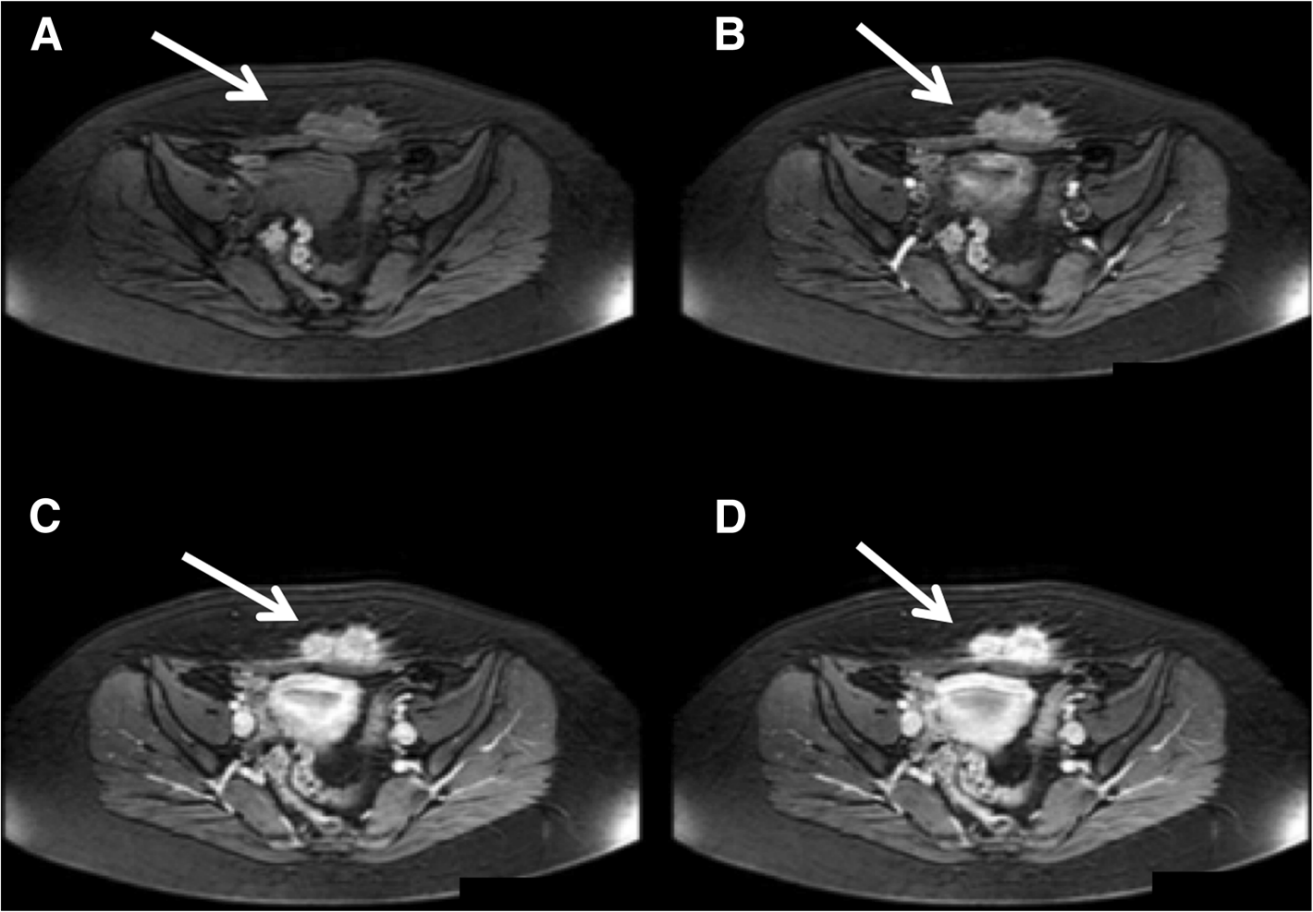
(Same patient of Fig. 11). Abdominal wall endometriosis (white arrows) showed progressive and late contrast enhancement on T1-THRIVE WI (**b**, arterial, **c**, portal and **d**, venous phases). **a**, T1-THRIVE WI before contrast administration

US is the first level examination and demonstrates a round or oval, heterogeneously hypoechoic solid lesion in the subcutaneous fat, muscle, or fascial layers. However, MR imaging is the modality of choice to evaluate the extension of disease because of its superior soft-tissue contrast and its capability to detect deep endometriosis. On MR, the typical lesion contains areas of T1 hyperintensity from subacute blood products. Depending on major content of fibrous tissue, as well as compact smooth muscle, some lesions may have an intermediate-to-low signal intensity on T1-WI and on T2-WI (Fig. 11) [26]. T1-WI after contrast administration typically shows late and progressive contrast enhancement (Fig. 12).

A rare differential diagnosis and cause of mass at the level of the abdominal wall in young women after cesarean delivery is the abdominal wall desmoid tumor (about 3.7 new cases occurring per one million individuals each year) [27]. These are rare, slow-growing benign muscular-aponeurotic fibrous tumors with the tendency to be locally aggressive.

**Fig. 13:**
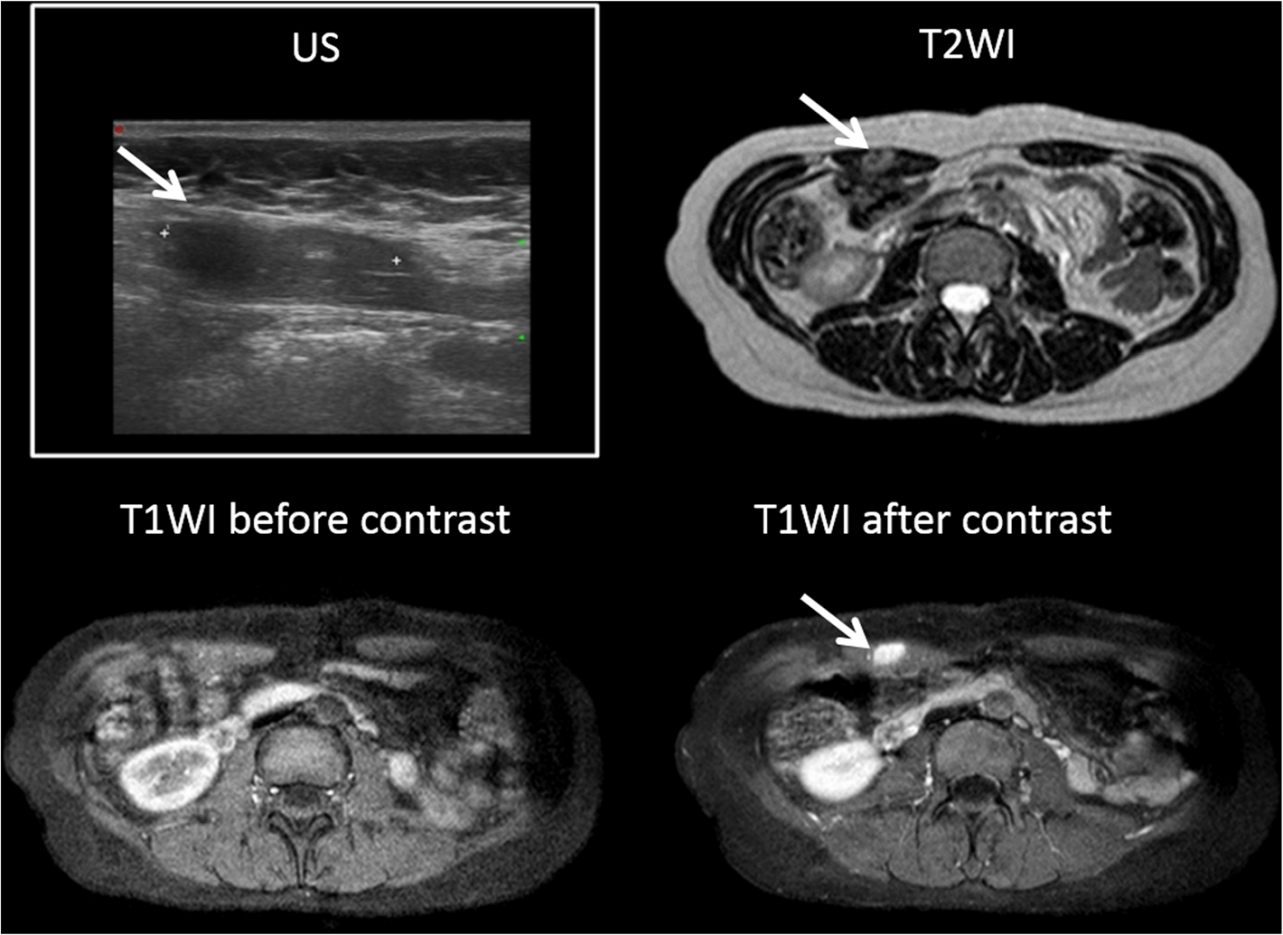
Desmoid tumor (white arrow) appears as homogeneously hypoechoic masses at US examination. On MR, typical signal characteristics include T1-WI and T2-WI low signal intensity and variable contrast enhancement (homogeneous in this case)

Surgical trauma is an important trigger for tumor growth as well as hormonal estrogenic influence [27, 28]. The sub-umbilical sheath of the rectus abdominis is the most common site (Fig. 13).

De Cian et al. also described a desmoid tumor arising in a cesarean scar during pregnancy [29].

Maybe due to its rarity, desmoid tumor has not been considered yet a chronic cesarean delivery complication. But radiologist must keep in mind this possible differential diagnosis for abdominal wall mass that develops during the postpartum period within 3 years after delivery.

**Cesarean scar ectopic pregnancy** is the implantation of the embryo in the cesarean delivery scar and it is the rarest form of ectopic pregnancy [1]. Estimated incidence in overall cesarean delivery is 1/1800–1/2500 [30]. Any process that disrupts or scars the endometrium and myometrium can predispose to abnormal pregnancy implantation.

Complications are severe, like uterine rupture and hemorrhage; these usually occur early in the pregnancy necessitating hysterectomy and occasionally resulting in death [31].

**Fig. 14:**
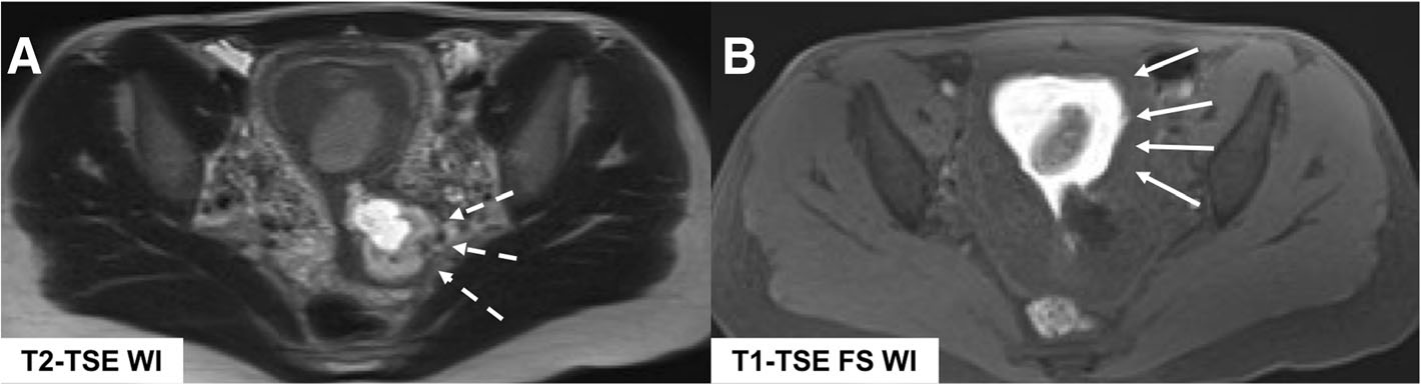
Cesarean scar ectopic pregnancy MR: T2-WI (**a**) show the gestational sac embedded in the myometrium of the anterior cervix–lower uterine segment (in this case antero-lateral, dashed arrow). T1-WI (**b**) demonstrates endometrial cavity distension by blood with endoluminal clots (white arrows) and the continuity between endometrial cavity and gestational sac itself

Early diagnosis is crucial to preserve fertility and reduce mortality. US is always the first level technique, whereas MR plays a crucial role in difficult cases (Fig. 14).

Imaging shows an empty uterine-cervical cavity and the gestational sac located predominantly in the lower uterine segment myometrium between the bladder and the anterior uterine wall.

**Retained products of conception** are estimated approximately 1% of term pregnancies [32].

The integration of clinical and ultrasonographic data is essential for diagnosis. RPOC can occur at the cesarean delivery scar and can be visualized at US as an irregular saclike remnant, an echogenic mass, or a mixed, solid, and cystic mass. However, the most accurate sign is trophoblastic low-resistance high-velocity arterial flow on color and pulsed Doppler US images [33]; in this study, the authors used both transabdominal and TV-US.

**Morbidly adherent placenta** (MAP) (placenta accreta, increta, and percreta) is abnormal placental invasion into the uterine wall, leading to failure of placental separation at delivery [34–41]. The incidence of morbidly adherent placenta has increased, with recent estimates approximating 1/333–1/533 deliveries [42, 43].

**Fig. 15:**
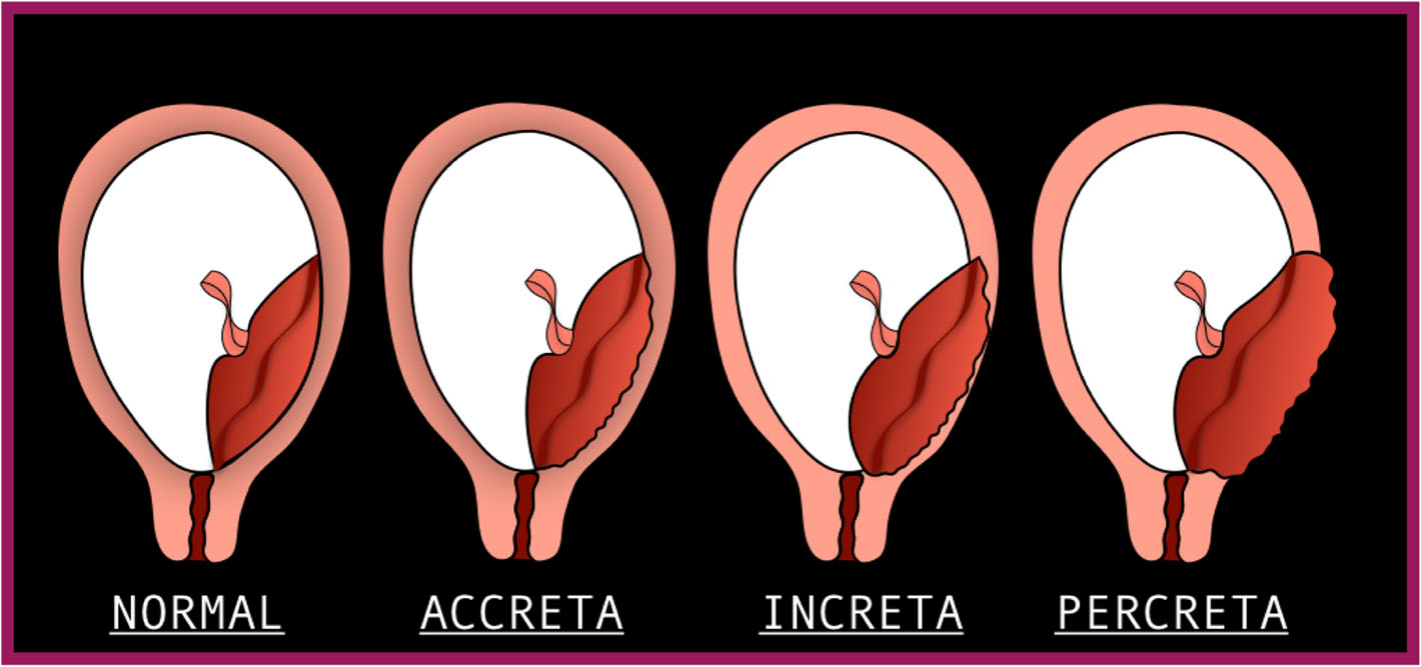
Morbidity adherent placenta classification

MAP is classified according to the depth of placental invasion into the uterine wall (Fig. 15):

• Accreta, the placenta is in direct contact with the myometrium

• Increta, the placenta invades into the myometrium

• Percreta, the placental invasion extends beyond the uterine serosa and into surrounding structures

US is always the first level technique while MR is useful in difficult cases.

Several main sonographic features of invasive placentation have been identified [44]:

- Direct visualization of placental tissue beyond the uterine cavity, such as a bulging mass in the urinary bladder (rare finding with low sensitivity but high specificity)
- Abnormalities of the placental–uterine interface, using grayscale ultrasound, such as loss of the normal hypoechoic retroplacental space
- Reduced lower-segment myometrial thickness
- Abnormal color Doppler findings identified as hypervascularity/abnormal vascularity of serosa–bladder interface, hypervascularity of uterine serosa–bladder interface, irregular intraplacental vascularization with tortuous confluent vessels across placental width
- Abnormal placental echostructure due to placental lacunae
- Parametrial invasion through a previous uterine scar

**Fig. 16:**
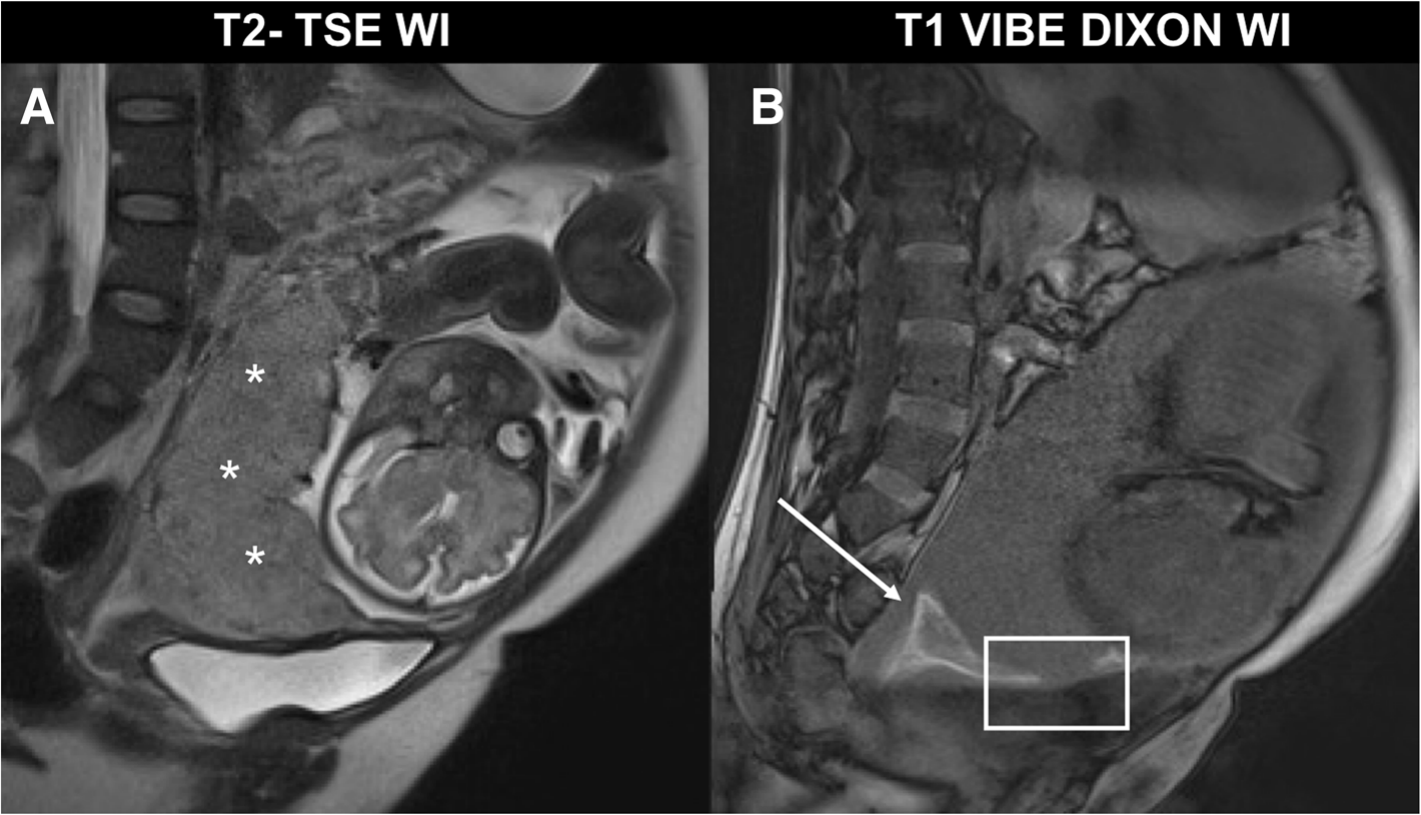
Morbidity adherent placenta: placenta previa (asterisk in **a**), cervical hematoma (white arrow in **b**) and placenta invasion into the myometrium (placenta increta, white box)

**Fig. 17:**
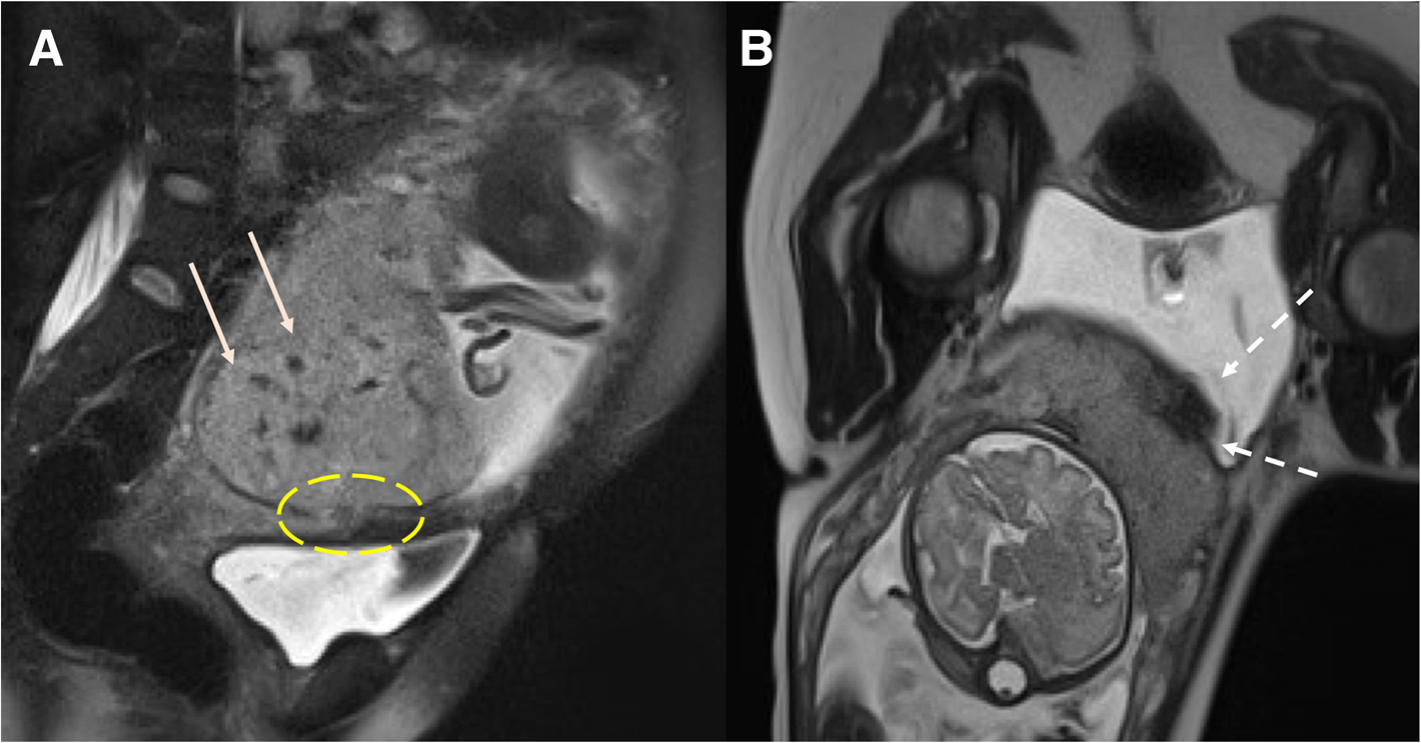
Morbidity adherent placenta MR findings on T2-WI (**a**, sagittal,Scientific Rep **b**, coronal): dark intraplacental bands (white arrows in **a**), thinning or loss of the retroplacental dark zone (round dashed box in **a**), and abnormal uterine bulge (dashed arrows in **b**)

MR features of MAP are dark intraplacental bands on T2-WI; abnormal uterine bulge, thinning, or loss of the retroplacental dark zone on T2-WI; myometrial thinning or focal disruption of the myometrium; heterogeneous placenta; and the possible invasion of adjacent organs (bladder) (Figs. 16 and 17).

Another important feature is identification of abnormal vasculature with multiple confluent, predominately small, serpiginous flow-void structures extending [45]:

- Along the maternal surface of the placenta and the uterine serosa, so-called serosal vessel
- From the uterine surface to the vesical-uterine fatty interface or even into the bladder wall (“bladder vessel” sign)
- From the surface of the uterus to the parametrial fat, “parametrial vessel”

The two most important risk factors of MAP are prior cesarean delivery and placenta previa.

MAP may lead to uncontrolled postpartum hemorrhage necessitating an emergent postpartum hysterectomy. For this reason, its prompt recognition can avoid important consequences.

## Conclusions

Due to the increasing frequency of cesarean delivery, radiologists will encounter more often its acute and chronic complications. Among these complications, CSD is the most common but also the most often undiagnosed one.

Awareness of normal postprocedural findings (myometrial defect and small bladder flap hematoma) helps radiologist to detect significant complications, including major hematomas, uterine dehiscence, and rupture. Repeated cesarean sections and severe CSD represent a predisposing factor for severe complications (such as abnormal placental implantation), especially in subsequent pregnancies: to familiarize with these various pathologic conditions is crucial to alert referring clinicians for a prompt and appropriate maternal and foetal management.

## Abbreviations

CSD: Cesarean scar defect
C-section: Cesarean-section
CT: Computed tomography
MAP: Morbidly adherent placenta
MR: Magnetic resonance
RPOC: Retained products of conception
TV-US: Transvaginal-ultrasound
US: Ultrasound
WI: Weighted imaging
